# Of mice and men: a comparative study of cancer-associated fibroblasts in mammary carcinoma

**DOI:** 10.3109/03009734.2012.658973

**Published:** 2012-04-19

**Authors:** Pernilla Roswall, Kristian Pietras

**Affiliations:** Department of Medical Biochemistry and Biophysics, Division of Vascular Biology, Karolinska Institutet, Stockholm, Sweden

**Keywords:** Biomarkers, breast neoplasms, fibroblast

## Abstract

**Introduction.:**

The initial clinical experience from targeted therapy for breast cancer has been mixed. While important progress has been made in the care of a subset of patients characterized by amplification of HER2 through the use of trastuzumab, other targeted therapies have failed to improve the outcome for large, unselected groups of patients. Thus, efforts to find prognostic or predictive biomarkers to enable tailored therapy are highly warranted. Genetically engineered mouse models of human cancer provide a convenient setting in which to perform explorative studies. However, there is a paucity of comparative studies between mouse and human tumours in order to validate the use of mouse models as discovery tools.

**Materials and methods.:**

Here, we have compared the localization of markers for cancer-associated fibroblasts in the MMTV-PyMT mouse model of mammary carcinoma with that of human breast cancer. The expression of α-smooth muscle actin, platelet-derived growth factor receptor-α, and fibroblast-specific protein-1 was assessed by immunostaining of sections from tumours of MMTV-PyMT mice. Information about the distribution of the same markers in human breast cancer was derived from the publicly available database the Human Protein Atlas.

**Results.:**

Both mouse and human mammary carcinomas were infused by a rich fibrotic stroma. While no marker was capable of identifying all stromal fibroblasts, the expression pattern of each marker was remarkably similar in mouse and human.

**Discussion.:**

We conclude that the MMTV-PyMT mouse model of breast cancer will have utility as a discovery tool for biomarkers of cancer-associated fibroblasts during malignant conversion.

## Introduction

The recent development of targeted therapy for cancer has been introduced into clinical practice over the course of the past decade. High hopes were placed on targeting specific over-active signalling pathways within tumours following the preceding successful drug development in the preclinical setting. Indeed, the therapeutic efficacy observed in mouse models of cancer has been translated into clinical benefit for patients. As a case in point, the humanized antibody trastuzumab (Herceptin), targeting the receptor tyrosine kinase HER2 in breast cancer, improves disease-free survival in HER2^+^ patients by 52% when administered in the adjuvant setting and prolongs overall survival by 24% for patients with metastatic disease (1,2). Nevertheless, many targeted therapies have failed to provide substantial improvement in survival in large phase III clinical trials. The main reason for this shortfall is a lack of reliable biomarkers predictive of response to therapy. In the few cases where robust biomarkers have been identified, such as HER2 amplification for trastuzumab treatment of breast cancer and lack of K-ras mutations for erlotinib treatment of non-small cell lung cancer (3,4), patient subsets that benefit significantly can be readily distinguished. In order to facilitate the discovery and genome-wide analysis of biomarkers for different subsets of cancer during the multistage malignant progression, the use of disease-specific genetically engineered mouse models of cancer with proven translational capacity will be increasingly valuable. Thus, there is a growing need for comparative studies between human and mouse tumours in order to validate preclinical models as surrogates for explorative studies aimed at finding new biomarkers and/or targets for cancer therapy.

Tumours are communicating organs. During the course of malignant conversion, cancer cells engage multiple different neighbouring cell types in the stroma; paracrine signalling between endothelial cells, pericytes, cancer-associated fibroblasts (CAFs), immune cells, thrombocytes, and the extracellular matrix provides a setting in which the tumour and stroma compartments co-evolve into a clinically manifested disease (5,6). As a rich provider of factors that collectively sustain tumour cell proliferation, survival, invasion, metastatic dissemination, and angiogenesis, the tumour stroma is currently an underestimated source of therapeutic targets and biomarkers with prognostic and predictive potential.

We set out to validate the use of a genetically engineered mouse model of breast cancer in the discovery of new biomarkers and therapeutic targets derived from CAFs. Pre-malignant lesions from MMTV-PyMT mice were found to be infused with CAFs already at an early stage. In overt invasive carcinomas, the marker-specific distribution and abundance of CAFs were remarkably similar to that of the human counterpart. Thus, we conclude that the MMTV-PyMT mouse model of breast cancer may have utility as a discovery tool for studies of CAFs during the malignant conversion of mammary carcinomas.

## Material and methods

### Mice and tumour samples

FVB/N-Tg(MMTV-PyVT)^634Mul/J^ transgenic mice have been described previously (7) and were purchased from the Jackson Laboratory. Mice of different ages (6,11, and 14 weeks) were anaesthetized with Avertin (Sigma Aldrich, St Louis, MO) and then euthanized by heart perfusion with Hanks' balanced salt solution followed by 4% paraformaldehyde (PFA). The left cervical and thoracic mammary glands were excised and subjected to overnight fixation in 4% PFA before embedding in paraffin. All animal experiments were approved by the Ethical Committee for Animal Experiments (Stockholm Norra djurförsöksetiska nämnd, application N96/11).

### Immunohistochemical analysis

Paraffin-embedded mouse mammary tissues were sectioned in 5 µm and adhered to glass slides, deparaffinized, and boiled in EnVision^™^ FLEX Target Retrieval Solution (DAKO, Glostrup, Denmark) for 20 min. Slides were incubated in 10% goat serum in TNBblocking solution (Perkin-Elmer Life Sciences, Waltham, MA). Antibodies were diluted in blocking buffer and incubated overnight; mouse anti-α-SMA (C-6198, Sigma Aldrich, St Louis, MO), rabbit anti-PDGFRα (#3164, Cell Signalling Technology, Danvers, MA), and rabbit anti-FSP1 (a kind gift from Dr Eric Neilson, Vanderbilt University, TN). After 1-h incubation with biotinylated secondary antibodies in blocking solution, the slides were subjected to an amplification step using Vectastain ABC kit (PK-6100 Standard, Vector Laboratories, Burlingame, CA). Peroxidase Substrate Kit DAB (Vector Laboratories) was used to develop and visualize the stainings. Slides were counterstained with Harris hematoxylin, dehydrated and mounted using X-TRA KITT (Medite Medizintechnik, Burgdorf, Germany). Images were taken using a Zeiss Observer Z1 microscope and an AxioCam HRc Zeiss camera.

## Results

**Figure 1.**
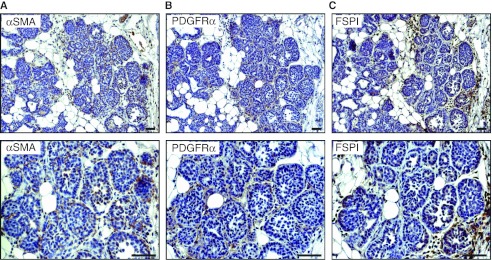
Expression of fibroblast markers in 6-week-old MMTV-PyMT mice. Representative immunostaining of hyperplastic lesions of a 6-week-old MMTV-PyMT mouse showing the expression pattern of ASMA (A), PDGFRα (B), and FSP1 (C). Scale bar is indicated in the figure and represents 50 µm.

The MMTV-PyMT mouse is a prototypical genetically engineered mouse model for multistage development of invasive ductal carcinoma of the breast (7). Tumours from MMTV-PyMT mice have previously been thoroughly characterized both genetically and histologically in terms of the tumour cell compartment compared to the human counterpart (8-10). However, as of yet, no comprehensive evaluation of the marker expression of CAFs has been reported. We have compared the expression and distribution of three widely used markers for CAFs and myofibroblasts—α-smooth muscle actin (ASMA), platelet-derived growth factor receptor-α (PDGFRα), and fibroblast-specific protein-1 (FSP1)—in tumours from MMTV-PyMT mice of different ages and human breast cancers. At 6 weeks of age, MMTV-PyMT mice present with small hyperplastic lesions or mammary intra-epithelial neoplasias (Figure 1). Immunostaining for ASMA, PDGFRα, and FSP1 revealed that each of the CAF markers is expressed by spindle-shaped stromal cells infiltrating into the mass of epithelial cells already at this early stage of tumour development (Figure 1A–C). None of the markers identified all stromal fibroblasts, indicating that there are different subsets of CAFs recruited into tumours (Figure 1A–C). Notably, ASMA was also expressed by myoepithelial cells that surrounded some of the tumour cell nests, as expected (data not shown).

**Figure 2.**
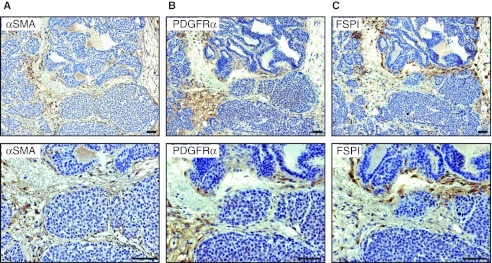
Expression of fibroblast markers in 14-week-old MMTV-PyMT mice. Representative immunostaining of tumour tissue from a 14-week-old MMTV-PyMT mouse showing the expression pattern of ASMA (A), PDGFRα (B), and FSP1 (C). The staining pattern for ASMA and PDGFRα shows similar staining pattern where staining of spindle-shaped stromal cells could be seen. For FSP1 not only stromal streaks were stained but also some cells intermingled in the epithelial islets. Scale bar is indicated in the figure and represents 50 µm.

Next, we analysed the abundance and marker profile of CAFs harboured within tumours from 11- and 14-week-old MMTV-PyMT mice. No major differences were noted in the expression pattern of ASMA, PDGFRα, or FSP1 between early and late tumours, even though the areas of fibroblast-rich stroma increased in size over tumour progression (data not shown). At 14 weeks of age, MMTV-PyMT mice presented with overt carcinomas infused with a fibroblast-rich stroma (Figure 2). Again, there was no marker capable of identifying all CAFs. Immunostaining for ASMA and PDGFRα yielded a similar pattern, with positive cells being predominantly elongated and located within streaks bridging nests of tumour cells (Figure 2A, B). In contrast, while still being located within fibrotic streaks surrounding tumour cell clusters, most FSP1^+^ cells were found to be rounded in shape (Figure 2C). In addition, a fraction of the cells expressing FSP1 was also interspersed as single cells between epithelial cells (Figure 2C).

**Figure 3.**
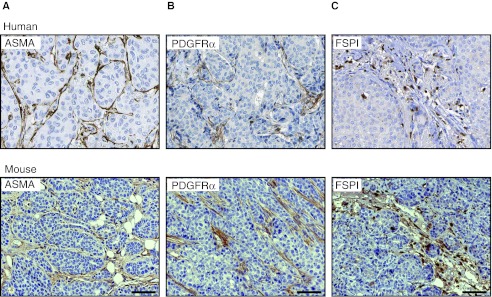
Correlation between CAF markers in mouse and human breast cancer. Immunostainings for ASMA (A), PDGFRα (B), and FSP1 (C), show a similar expression pattern in human (upper panel) and mouse (lower panel) mammary tumour tissue. Scale bar is indicated in the figure and represents 50 µm.

To investigate the correlation between CAF marker expression in tumours from MMTV-PyMT mice and human breast cancer, we analysed the abundance and phenotype of cells marked by ASMA, PDGFRα, and FSP1 using publicly available data from the Human Protein Resource (see: http://www.proteinatlas.org) (11). The expression of each CAF marker in human tumours was heterogenous and varied from patient to patient. Nevertheless, the consensus staining pattern for ASMA, PDGFRα, and FSP1 was remarkably similar to that of the tumours from MMTV-PyMT mice (Figure 3A–C). Thus, cells expressing ASMA and PDGFRα were located in fibrotic streaks and displayed a spindle-shaped morphology (Figure 3A, B), whereas FSP1^+^ cells were distinguished by a rounded shape and located both in the overt stroma and within tumour cell clusters (Figure 3C).

In summary, none of the markers used in this study was able to identify all subsets of CAFs. However, striking similarities between mouse and human breast tumours were found in terms of the distribution and morphology of stromal fibroblasts expressing ASMA, PDGFRα, and FSP1, respectively.

## Discussion

Previous comparisons between tumours from genetically engineered mouse models and human breast cancer have mainly focused on the tumour cells (12). Lesions from the initial stages of malignant transformation of the MMTV-PyMT mouse have been found mostly to resemble human breast cancers of the luminal B subgroup with expression of ER/PR and HER2 as common denominators (9). With increasing malignancy, mouse tumours tend to lose expression of hormone receptors, while HER2 expression escalates. Indeed, tumour shrinkage can be readily induced in MMTV-PyMT mice by treatment with trastuzumab (K.P., unpublished observation). Evaluations of the human resemblance of the stromal compartment of tumours from the MMTV-PyMT mouse are scarce, even though it was noted in a pathologist consensus report on mouse models of mammary carcinoma that these tumours harbour a densely sclerotic stroma (12). Here, we have contrasted the localization and abundance of three different markers for CAFs in tumours from MMTV-PyMT mice with human breast cancer. As noted in previous studies (13-15), no marker labelled all stromal fibroblasts, suggesting that different subsets of CAFs populate the tumour stroma already at an early stage. While it is unclear whether different subtypes of CAFs represent different functionalities, various subsets may also reflect different origins, since CAFs have been reported to be recruited into tumours from both local and remote sources (14,16). Of note, tumours from the MMTV-PyMT mouse model faithfully reproduced the staining pattern of the CAF markers ASMA, PDGFRα, and FSP1 found in human tumours. Thus, the close similarity between mouse and human cancer in this aspect lends support to the notion of using genetically engineered mice in the discovery process of CAF-derived therapeutic targets and biomarkers.

The pursuit of predictive biomarkers will be imperative to enable tailored targeted therapy to patients. However, the search for such markers should not be confined to the tumour cell compartment. The pre-malignant epithelium and surrounding stromal compartment continuously fertilize each other during the malignant conversion. Thus, molecular changes indicative of prognosis and/or therapeutic response may arise early during tumour progression. Indeed, it may even be that the metastatic microenvironment is shaped already before tumour cells are present, thereby raising the possibility that changes manifested in the stroma of would-be metastatic organs can serve as useful markers for preventive measures (17). We found that stromal fibroblasts expressing ASMA, PDGFRα, and FSP1 readily infiltrate early pre-malignant lesions from MMTV-PyMT mice, again indicating that CAFs are particularly interesting targets for the discovery of biomarkers also for early stages of the disease.

Genome-wide efforts have recently identified stromal gene expression signatures with prognostic or predictive capabilities in breast cancer (18,19). Finak et al. identified a 26-gene stroma-derived signature that distinguished between good, poor, and mixed survival in three independent data sets of more than 750 breast cancer patients (19). Likewise, a second gene signature from the tumour stroma was found to hold predictive power for the outcome of neoadjuvant chemotherapy in breast cancer (18). These studies provide proof of principle that the stromal compartment of tumours influences the prognosis and outcome of the disease. However, the breast tumour microenvironment contains a mixed population of cells, including fibroblasts, endothelial cells, pericytes, immune cells, and adipocytes, and information may thereby be diluted when considering the total gene expression profile of the stroma. Thus, efforts to fractionate tumours into different cellular compartments and analyse the undiluted and isolated cell types for prognostic or predictive biomarkers may hold greater utility. The use of CAFs in this regard is highly motivated by their proven capability of promoting tumour initiation and growth (5). We conclude that the close similarities between CAFs in mouse and human tumours, as elucidated in the current study, provide a valuable platform for cross-validation of newly discovered therapeutic targets and biomarkers expressed by CAFs.
